# A Comparative Metabolomics Approach Reveals Early Biomarkers for Metabolic Response to Acute Myocardial Infarction

**DOI:** 10.1038/srep36359

**Published:** 2016-11-08

**Authors:** Sara E. Ali, Mohamed A. Farag, Paul Holvoet, Rasha S. Hanafi, Mohamed Z. Gad

**Affiliations:** 1 Department of Pharmaceutical Biology, Faculty of Pharmacy & Biotechnology, The German University in Cairo, Egypt; 2 Department of Pharmacognosy, Faculty of Pharmacy, Cairo University, Cairo, 11562, Egypt; 3 Department of Cardiovascular Sciences, Atherosclerosis and Metabolism Unit, KatholiekeUniversiteit Leuven, Belgium; 4 Department of Pharmaceutical Chemistry, Faculty of Pharmacy & Biotechnology, The German University in Cairo, Egypt; 5 Department of Biochemistry, Faculty of Pharmacy & Biotechnology, The German University in Cairo, Egypt

## Abstract

Discovery of novel biomarkers is critical for early diagnosis of acute coronary syndrome (ACS). Serum metabolite profiling of ST-elevation myocardial infarction (STEMI), unstable angina (UA) and healthy controls was performed using gas chromatography mass spectrometry (GC/MS), solid-phase microextraction coupled to gas chromatography mass spectrometry (SPME-GC/MS) and nuclear magnetic resonance (^1^H-NMR). Multivariate data analysis revealed a metabolic signature that could robustly discriminate STEMI patients from both healthy controls and UA patients. This panel of biomarkers consisted of 19 metabolites identified in the serum of STEMI patients. One of the most intriguing biomarkers among these metabolites is hydrogen sulfide (H_2_S), an endogenous gasotransmitter with profound effect on the heart. Serum H_2_S absolute levels were further investigated using a quantitative double-antibody sandwich enzyme-linked immunosorbent assay (ELISA). This highly sensitive immunoassay confirmed the elevation of serum H_2_S in STEMI patients. H_2_S level discriminated between UA and STEMI groups, providing an initial insight into serum-free H_2_S bioavailability during ACS. In conclusion, the current study provides a detailed map illustrating the most predominant altered metabolic pathways and the biochemical linkages among the biomarker metabolites identified in STEMI patients. Metabolomics analysis may yield novel predictive biomarkers that will potentially allow for an earlier medical intervention.

Despite considerable advances in the treatment of acute coronary syndrome (ACS), it remains the leading cause of morbidity and mortality worldwide^1^. Recognition of myocardial ischemia is critical for both assessing the outcome of ACS and evaluating the response to therapeutic interventions. It is possible to accurately diagnose patients with irreversible injury secondary to myocardial infarction (MI) using several biomarkers. However, none are suitable for detecting the more subtle insult of myocardial ischemia^2^. This lack of suitable biomarkers prevents the detection of early cardiovascular disease (CVD) risk conditions, and hampers timely and effective risk assessment, prevention and management.

Novel biomarkers that can facilitate interventions to prevent the progression of the disease to a severe form are desired and needed. This will reduce the use of unnecessary resources in the workup of patients and avoid inappropriate discharges^3^. In this scenario, biomarker profiles with the ability to reliably discriminate ischemic from non-ischemic patients would be of inordinate value, and could have important clinical implications in daily practice.

Many of the commonly accepted CVD risk factors, such as abdominal obesity^4^ and insulin resistance^5^, have a metabolic origin. Moreover, altered cardiac metabolism is the primary consequence of myocardial ischemia^3^. Metabolite levels change rapidly in response to physiologic perturbations as they represent proximal reporters of disease phenotypes^6^. The analysis of low-molecular-weight blood metabolites can indeed offer a “fingerprint” of the underlying biophysical system and provide insights into the biochemical processes and their regulation^7^.

Metabolomics permits a quantitative measurement of the multivariable metabolic responses of living systems to pathophysiological stimuli. This is achieved by simultaneously monitoring changes in hundreds of low-molecular-weight metabolites in tissues or biofluids^6^. Due to the complexity of the metabolome and metabolites diverse properties, no single analytical method can be used to analyze all the metabolites in a biological sample^8^. Several metabolomics platforms have been employed for metabolome measurement. Among several detection methods, nuclear magnetic resonance (NMR) spectroscopy and mass spectrometry (MS); coupled to an array of separation techniques, including gas chromatography [GC] or liquid chromatography [LC] are the two most common technologies that prevail as the workhorses for analysis of biological samples^9^. Integrated analytical techniques have frequently been used to enable the sensitive and reliable detection of hundreds of metabolites in serum; in addition to accelerating the integration of metabolomics into disease diagnostics research^10^.

A number of targeted and untargeted strategies have been developed for metabolomics analyses^6^. The targeted approach relies on the analysis of a set of pre-defined metabolites in the samples of interest. Although this approach provides high sensitivity, precision and accuracy due to the use of stable isotope internal standards, it covers only a part of the metabolome. The untargeted metabolomics approach mostly involves the unbiased analysis of a large number of metabolites^11^. Such an approach provides greater coverage of the metabolome and is commonly utilized at the initial stages of the biomarker discovery process and later to be confirmed *via* other targeted profiling methods^12^.

The current study presents an untargeted comparative metabolomics approach using multiplatform MS and NMR high-throughput analytical technologies. The objective was to provide insights into the underlying metabolic pathways that are perturbed in two cardiovascular pathologies: unstable angina (UA) and ST-elevation myocardial infarction (STEMI). Here, we attempted to establish a metabolic signature of myocardial injury in order to identify predictive biomarkers that will potentially allow for an earlier intervention and/or a more effective approach to treatment. In addition, a targeted enzyme-linked immunosorbent assay (ELISA) was used to further quantify the absolute levels of one of the most intriguing molecules in the current results.

## Results

### GC/MS-based metabolite profiling and multivariate data analyses

**Figure 1:**
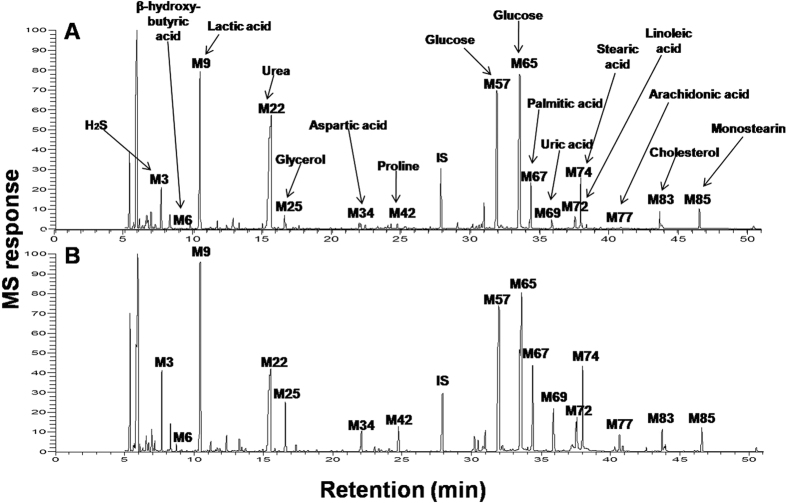
Representative GC/MS chromatograms of serum derived from a healthy control (**A**) and a STEMI patient (**B**). Peak numbers correspond to those listed in (Supplementary Table ST1).

A total of 68 metabolites were identified in serum samples from STEMI patients, UA patients and healthy controls using GC/MS. The identity, retention time (rt), retention index (RI) and mass-to-charge ratio (*m/z*) of compounds are shown in Supplementary Table S1. Peaks were identified and attributed to endogenous metabolites that are known to be involved in biochemical processes, especially in energy and lipid metabolism^13^. These included organic acids, amino acids, fatty acids, sugars and signaling gasotransmitters. Representative GC/MS chromatograms showing the average peaks from healthy controls and STEMI patients are depicted in (Fig. 1). Differences in the peak intensities were observed among the two groups, with major variant peaks belonging to hydrogen sulfide (H_2_S), glycerol, lactic acid, uric acid and fatty acids.

**Figure 2:**
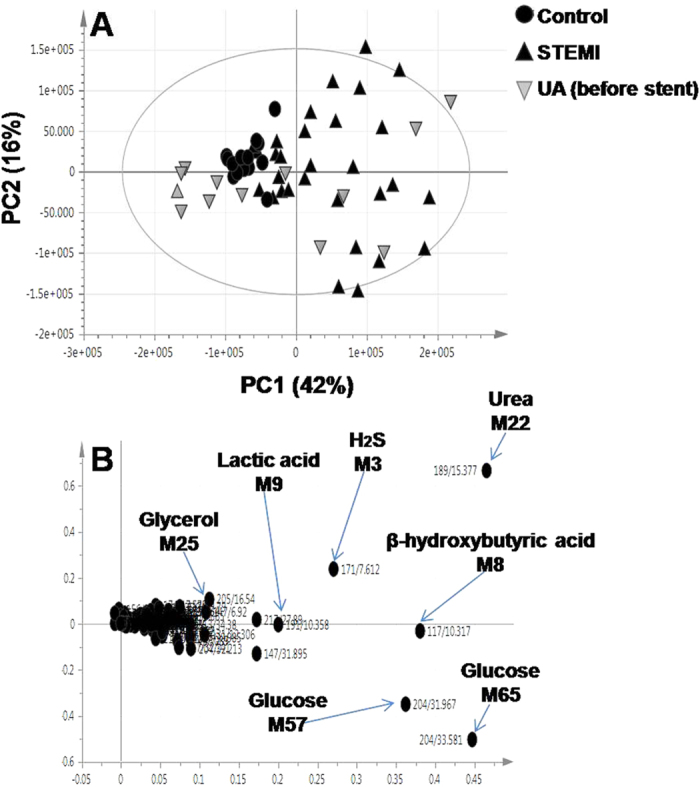
GC/MS based PCA of STEMI patients (▴), before stent samples of UA patients (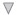) and healthy controls (●) (**A**) Score plot of PC1 and PC2 scores (**B**) Loading plot for PC1 components contributing peaks and their assignments, with each metabolite denoted by its mass/rt (min) value. Peak numbers correspond to those listed in (Supplementary Table ST1).

The acquired data were complex as a result of the large number of monitored metabolites. In order to better visualize the subtle similarities and differences among these complex datasets, multivariate data analyses, i.e., supervised and unsupervised methods were employed. The unsupervised analysis methods as principal component analysis (PCA) was applied to the GC/MS dataset to reduce the dimensionality of the data while retaining most of the variation in the dataset^14^. The PCA score plot shows a clear separation between STEMI patients and healthy controls, while the differences between before stent samples of UA patients and healthy controls were not so clear (Fig. 2A). The first two components (PC1 and PC2) explained 42% and 16% of the total variance, respectively. The corresponding loading plot of PC1 indicated that serum of STEMI patients exhibited higher levels of H_2_S, β-hydroxybutyric acid, lactic acid, urea, glycerol and glucose as compared to healthy controls (Fig. 2B). Therefore, these metabolites may be regarded as marker metabolites for the STEMI group.

**Figure 3:**
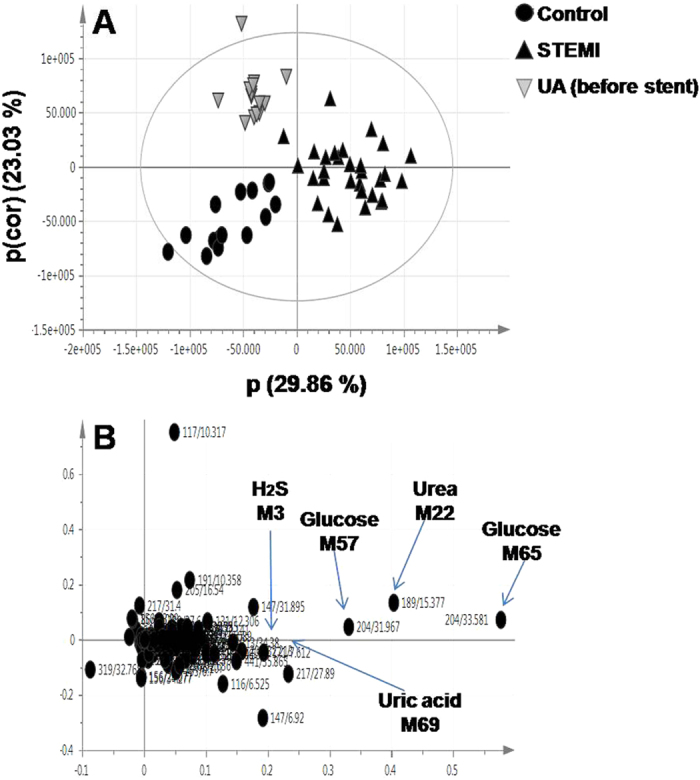
GC/MS based OPLS-DA of STEMI patients (▴), before stent samples of UA patients (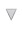) and healthy controls (●) (**A**) OPLS-DA score plot (**B**) loading plot derived from samples modeled against each other. Selected variables are highlighted in the loading plot with each metabolite denoted by its mass/rt (min) value. Peak numbers correspond to those listed in (Supplementary Table S1).

A supervised method as orthogonal projection to latent structures-discriminant analysis (OPLS-DA) was used to improve separation between groups^14^. The OPLS-DA score plot demonstrates clear separation between STEMI patients and healthy controls and to a lesser extent between before stent samples of UA group and healthy controls (Fig. 3A), as in agreement with the PCA score plot (Fig. 2A). The axes plotted in the S-plot represent the covariance p[1] against the correlation p(cor)[1]. The loading plot shows an increase in the levels of H_2_S, urea, uric acid and glucose in the serum of STEMI patients (Fig. 3B).

This study also aimed to detect other less abundant low molecular weight metabolites. Therefore, major metabolites revealed from the first multivariate analysis were excluded from the dataset. OPLS-DA was applied to this cut biased dataset in a second attempt. The OPLS-DA score plot for STEMI patients versus healthy controls shows a distinct separation between the two groups (Supplementary Fig. S1A), as in agreement with the first OPLS-DA model (Fig. 3A). As compared to healthy controls, a number of low molecular weight metabolites showed increased concentration in the serum of STEMI patients, such as α-hydroxyisobutyric acid, valine, palmitic acid and uric acid, while citrulline was observed in decreased levels (Supplementary Fig. S1B).

### GC/MS-based fatty acids profiling and multivariate data analyses

Serum lipids and lipoproteins usually undergo several phased changes in response to MI. Moreover, free fatty acids (FFA) concentration increases precipitously during the early-onset MI^15^. Therefore, the potential of using various lipids fractions as biomarkers for predicting the risk of MI was tested. The current study specifically aimed at investigating the changes in the levels of fatty acids and cholesterol in response to the disease. A total of 12 fatty acids and cholesterol were identified in serum samples from STEMI patients, UA patients and healthy controls using GC/MS (Supplementary Table S1), where they were separately subjected to multivariate data analysis. The PCA score plot shows two clusters of samples relating to healthy controls and STEMI patients, while before stent samples of UA patients were overlapping with healthy controls (Supplementary Fig. S2A). This suggests that there is a dynamic change in the fatty acids profile of STEMI patients. The fatty acids that most contributed for such segregation were palmitic acid, linoleic acid, stearic acid and oleic acid, being relatively elevated in the serum of STEMI patients, whereas no significant difference was observed for the level of cholesterol among different groups (Supplementary Fig. S2B). These results confirm that MI induced marked changes in the levels of FFA.

OPLS-DA analysis was also performed on the fatty acids profile of these patients. The OPLS-DA score plot shows a clear separation between STEMI patients and healthy controls, whereas before stent samples of UA patients were still overlapping with healthy controls (Supplementary Fig. S3A). The corresponding loading plot confirmed the elevation of palmitic acid in STEMI patients (Supplementary Fig. S3B), as identified above.

### The effect of coronary stenting on the GC/MS derived metabolite profiles of UA patients

**Figure 4:**
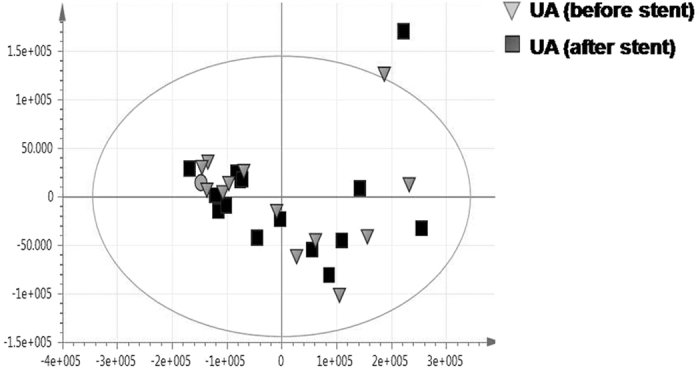
GC/MS based PCA of before stent (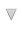) versus after stent samples (■) of UA patients.

In an attempt to investigate the effect of coronary stenting on the metabolite profiles of UA patients, a PCA model was performed for before stent versus after stent samples. The PCA score plot shows no discrimination between samples, indicating that coronary stenting had no clear effect on the metabolite profiles of UA patients. Samples were scattered on the score plot due to the variability in the severity of the disease within this cohort (i.e., lesion severity and size of the vessel being treated) (Fig. 4).

Moreover, no valid OPLS-DA model could be derived from modeling before stent versus after stent samples. This confirms that both the supervised and unsupervised analysis were not able to discriminate between samples pertaining to the UA patients.

### Quantitative determination of serum H_2_S using ELISA

**Figure 5:**
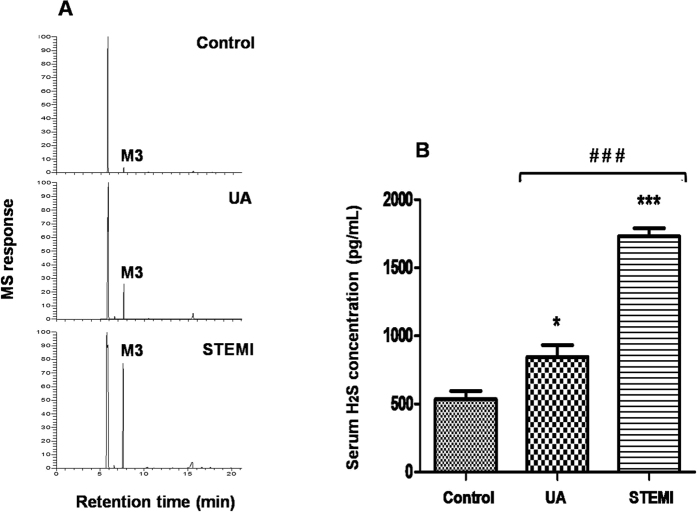
(**A**) Reconstructed GC/MS chromatograms for serum H_2_S in samples derived from a healthy control, UA patient and a STEMI patient illustrating the difference in H_2_S peak (M3) among different groups. Peak numbers correspond to those listed in (Supplementary Table ST1) (**B**) ELISA measured absolute H_2_S serum levels in samples from healthy controls, before stent samples of UA patients and STEMI patients. Results are expressed as mean ± SEM. Statistically significant difference was observed between UA patients and healthy controls (^*^P ≤ 0.05), STEMI patients and healthy controls (^***^P ≤ 0.001), and STEMI patients and UA patients (^###^P ≤ 0.001).

One of the most intriguing marker metabolites that indeed merit further investigation is H_2_S, an endogenous signaling gasotransmitter with profound effect on the heart and circulation^16^. H_2_S is one of the major discriminatory metabolites observed in the loading plots belonging to the STEMI group (Figs 2B and 3B). The considerable interest in H_2_S urged for further monitoring of its level using a double antibody sandwich ELISA. Reconstructed GC/MS chromatograms for serum H_2_S in samples derived from a healthy control, UA patient and a STEMI patient are shown in Fig. 5A, illustrating the difference in the level of H_2_S among different groups. This highly sensitive immunoassay confirmed the elevation of H_2_S in the serum of STEMI patients, as compared to UA patients and healthy controls. Similarly, the level of H_2_S was elevated in the serum of UA patients, as compared to healthy controls. However, the increase was less pronounced in UA patients than in STEMI patients. Thus, serum H_2_S level was able to discriminate between UA and STEMI patients (Fig. 5B).

### Headspace solid phase microextraction coupled to gas chromatography mass spectrometry (SPME-GC/MS) of serum volatile metabolites

The indication of anaerobic metabolism in STEMI patients prompted monitoring other volatile metabolites products of anaerobic metabolism which could have evaded detection using such GC/MS methodology. Only samples that showed the most variant response from GC/MS analysis of primary metabolites were chosen for SPME-GC/MS analysis.

SPME was attempted to analyze serum volatile metabolites without the prior need for derivatization and with trapped volatiles subsequently analyzed by GC/MS. Results of this sensitive technique show an increase in acetone levels in the serum of some STEMI patients as compared to healthy controls (Supplementary Fig. S4).

### ^1^H-NMR-based metabolite fingerprinting and multivariate data analyses

Another complementary technique, ^1^H-NMR, was applied to provide a broader range of metabolite coverage. A total of 52 metabolites were identified in serum samples from STEMI patients, UA patients and healthy controls using ^1^H-NMR. The less number of metabolites detected using NMR compared to 68 peaks *via* MS is attributed to MS higher sensitivity levels. The identities, chemical shift (δ), coupling constant (J) and multiplicity for individual components are presented in Supplementary Table ST2. A representative NMR spectrum of a healthy human serum is shown in Supplementary Fig. S5.

**Figure 6:**
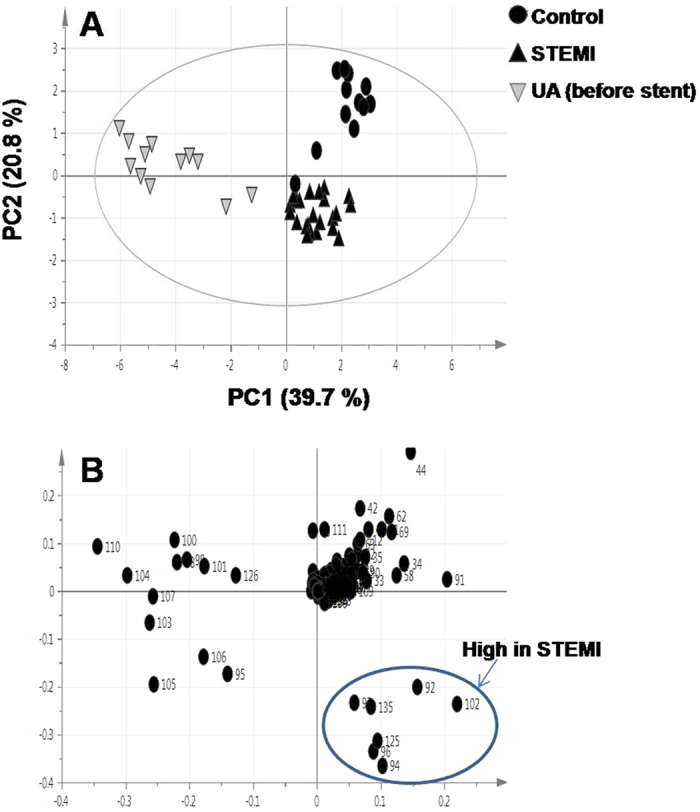
^1^H-NMR based PCA of STEMI patients (▲), before stent samples of UA patients (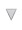) and healthy controls (●) (**A**) Score plot of PC1 and PC2 scores (**B**) Loading plot for PC1 components contributing bin numbers. Differential signals high in STEMI were assigned in each bin as follows: Bin 92, D-glucose, carnitine and betaine; bin 94, D-glucose; bin 96, choline and D-glucose; bin 97, D-glucose, glycerol and glycine;bin 102, D-glucose; bin 125, β-glucose; bin 135, α-glucose.

Multivariate data analysis was performed for the spectral region of δ −0.4 to 9.0 ppm. The binned data was initially subjected to PCA with the first two PCs accounting for 39.7% and 20.8% of the total variance, respectively. The PCA score plot shows three distinct clusters relating to STEMI patients, before stent samples of UA patients and healthy controls (Fig. 6A). The loading plot displays the variables (in bin numbers) responsible for the clear separation observed in the score plot. The corresponding loading plot of PC1 indicated increased levels of carnitine, betaine, choline, glycerol, glycine and glucose in the serum of STEMI patients (Fig. 6B).

A further PCA was performed for STEMI patients versus healthy controls, a PC score plot (PC1 = 40.8% and PC2 = 23.7%) shows a distinct separation between the two groups. However, these groups were clearly separated along PC2 (Supplementary Fig. S6A). The loading plot for PC2 exposed the most discriminatory signals and confirmed the elevation of choline, glycerol, glycine, glucose, lactic acid and β-hydroxybutyric acid in the serum of STEMI patients (Supplementary Fig. S6B).

The PCA models derived from the ^1^H-NMR analysis show that NMR signals belonging to lactic acid, α-glucose and β-glucose were the most significant in contributing to sample group separation (Fig. 6B and Supplementary Fig. S6B). Therefore, quantitative NMR analysis was performed for these metabolites. Results indicated that the estimated levels of these metabolites were higher in the serum of STEMI patients as compared to healthy controls (Supplementary Fig. S7).

A supervised OPLS-DA analysis was performed for STEMI patients versus healthy controls. Goodness of fit and predictive ability values (R^2^ and Q^2^) were 0.686 and 0.629, respectively. The OPLS-DA score plot shows a clear separation between the two groups (Supplementary Fig. S8A). The corresponding loading plot confirmed the increase in the levels of carnitine, betaine, choline, glycerol, glycine and glucose in STEMI group (Supplementary Fig. S8B). These data mirrored the PCA loading plot derived from the ^1^H-NMR analysis (Fig. 6B).

In summary, our data indicate that alterations in metabolism are dominated by the MI state with major discriminatory metabolites observed in all loading plots belonging to the STEMI group, whereas the UA-related changes in the profiles, although contributing to group separation, are less apparent (Figs 1, 2, 3, 4, 5 and 6 and Supplementary Figs S1–S8). The outcome of different metabolomics technologies is shown in (Tables 1 and 2). Biomarker metabolites identified in the serum of STEMI patients and their associated metabolic pathways are represented in Fig. 7.

## Discussion

Current markers for myocardial injury (i.e., Creatine kinase-MB (CK-MB) and cardiac troponin) are not reliably detected until at least 4–6 h post myocardial injury, and once detected; the disease is already in its irreversible state^17^. In contrast, the metabolic changes identified in the present study were readily apparent as early as 1–2 h post myocardial injury, a time frame in which to our knowledge, no currently used biomarkers are found to be elevated.

This study aimed at investigating early markers of endothelial and vascular dysfunction in an attempt to identify a disease status that has yet to become symptomatic. A multiplex comparative metabolomics approach including GC/MS, SPME-GC/MS and ^1^H-NMR was for the first time applied for a comprehensive metabolites assessment in two common forms of ACS, as STEMI and UA. Previous metabolomics studies have generally employed either NMR^3^ or GC/MS^18^. There is growing evidence that no single method is adequate and that combining both MS and NMR offers a powerful approach in leveraging between both technologies limitations.

In the current metabolomics study, UA patients were clinically stable and recruited at elected angioplasty. This may explain why the metabolite profiles of UA patients were not significantly different from healthy controls. Indeed, UA patients were not in the active phase at the time of blood sampling; rather they were treated because of earlier episodes of acute events. In addition, the metabolite profiles of UA patients were not altered after reperfusion which suggests that successful coronary angioplasty restores normal coronary circulatory dynamics^19^ (Fig. 4).

In STEMI patients, samples were taken from patients presenting at the triage and before admission to the ICU. Patients presented with severe chest pain accompanied by diaphoresis, nausea and sometimes syncope suggesting acute myocardial infarction (AMI). Serum collection was performed prior to the administration of any medications and when patients’ baseline troponin levels were normal. A dramatic and immediate change in the global metabolism of STEMI patients was observed as a consequence of insufficient blood flow and subsequent stress response. Serum metabolite profiling of STEMI patients revealed for a myriad of differentially occurring metabolites. These included fatty acids such as palmitic acid, stearic acid, linoleic acid and oleic acid, ketone bodies as acetone and β-hydroxybutyric acid, amino acids as valine, glycine, carnitine and citrulline, organic acids as α-hydroxyisobutyric acid, choline, betaine, lactic acid, uric acid, urea and glycerol, sugars as glucose, and signaling gasotransmitters as H_2_S. The identified metabolites present a detailed map of the metabolic pathways perturbed by MI and provide information about the metabolic state of myocardial tissue that is associated with the disease. The major recognized altered metabolic pathways are mainly referred to as oxidative stress and ischemia-induced alterations in energy metabolism, amino acids metabolism, fatty acid oxidation, anaerobic glycolysis, urea cycle and pathways linked to endogenous gasotransmitters. These metabolic changes could be useful for early risk stratification of MI patients, particularly if troponin results are negative upon patient hospital admission, as observed in STEMI patients (Table 3). Using such comparative metabolomics approach, we were able to assign distinct clusters of related metabolites that exhibit coordinate responses to ischemia and have great potential in the earlier diagnosis of STEMI patients.

Our data suggest that ischemia constitutes the main axis that drives the metabolism of each cell to adapt to the deficiency of oxygen in order to maintain their metabolic activity. Based on the ischemic insult severity, elevation of catecholamine levels can be observed for prolonged periods of up to 24 h^20^. Ischemic stress is also associated with elevation of hydrocortisone levels which can often blunt insulin sensitivity. Insulin resistance that develops in adipose tissue results in altered ability of insulin signaling cascade to store triglycerides. This will induce lipolysis and uncontrolled release of FFA and glycerol^21^,^22^,^23^. Consistent with such hypothesis, our results indeed points to elevated levels of glucose, glycerol and FFA as palmitic, stearic, linoleic, and oleic acid in the serum of STEMI patients (Fig. 2B and Supplementary Fig. S2B).

The metabolic alterations in myocardial energy production also affect other important metabolic pathways^3^. The decline in glucose oxidation during ischemia requires the rapid acceleration in the conversion of pyruvate to lactate in order to regenerate NAD^+^ under oxygen limiting conditions, which is required to sustain glycolysis^24^. In addition, an increased reliance on the anaerobic myocardial metabolism takes place, which increases lactic acid release out of cardiac myocytes for maintenance of ATP levels. The overall result is an increase in circulating lactate after ischemia^3^, as clearly evident from our results (Fig. 2B).

The elevated levels of ketone bodies is another common metabolic area that could also relate to stress on energy metabolism. Both β-hydroxybutyrate and acetone are ketone bodies that are mainly synthesized from the oxidation of fatty acids and are known for their roles in glucose and lipid metabolism^25^. In case of starvation or diabetes mellitus, the levels of ketone bodies are significantly increased due to high levels of fatty acids and low insulin. In this case, they become the major energy supplier of the myocardium^26^. Results of the present study concurring with others^18^ suggest that the hypoxia situation comes to “mimic” the physiological situation that occurs in diabetes. The low energy yield of glucose metabolism forces cells to use fat as an energy source and release ketone bodies as end products. Consequently, elevation in ketone bodies levels denotes for an increase in fatty acids catabolism concurrent with the incapacity of the TCA cycle to fully metabolize acetyl CoA^27^(Fig. 2B and Supplementary Fig. S4).

Another interesting potential biomarker that signals for either hyperglycemia or dysregulation of fatty acids metabolism is α-hydroxyisobutyric acid. It was also reported in the blood and urine of lactic acidosis patients^28^. The present study shows an increase in its level that is believed to arise from inefficient fatty acids oxidation (Supplementary Fig. S1B).

Carnitine is an essential metabolic mediator which facilitates β-oxidation by transporting the activated fatty acids into the mitochondrial matrix^29^. Accumulation of carnitine in the serum of STEMI patients may be indicative of an increased β-oxidation and mitochondrial dysfunction (Fig. 6B). Serum free L-carnitine in combination with CK-MB and myoglobin was suggested to be used as a predictor for the diagnosis of AMI^30^. Increase in carnitine and branched chain amino acids (BCAA) levels have been found to act as a biomarker of insulin resistance in CVD patients^31^.

The principal end product of protein catabolism, urea, was found to be increased in the circulation of MI group (Fig. 2B). In contrast, citrulline was detected at much lower levels as compared to healthy controls (Supplementary Fig. S1B), both of which are members of the urea cycle that feeds into the citric acid cycle. Therefore, alteration of their levels indicates that urea cycle has also been impaired^32^. Sabatine *et al*. group^33^ observed a drop in the level of citrulline in AMI patients. Such decrease in citrulline levels is likely to be mediated *via* the preservation of the citric acid cycle intermediates to defend ATP production in the myocardium.

Oxidative stress has been regarded as one of the most important contributors to the progression of atherosclerosis^34^. An interesting finding revealed from the current results that could relate to oxidative stress, is uric acid. During periods of oxidative stress, mitochondria become less capable in converting ADP to ATP. This results in ADP to be shunted to the production of hypoxanthine which is associated with an increase of uric acid^35^, as observed in STEMI patients (Fig. 3B). Uric acid is regarded as a potential risk factor for the development of CVD^36^.

Changes in serum phospholipids levels are associated with silent myocardial ischemia^37^. Griffin *et al*.^38^ reported that the main metabolic changes monitored using NMR in CVD patients were derived from lipids in lipoproteins, as well as choline. Moreover, Senn *et al*.^39^ reported an increase in choline and betaine levels in patients undergoing coronary angiography. Such an upregulation in these metabolites, being linked *via* a common biochemical pathway, add credence to the current results reported herein for observed choline and betaine increased serum levels (Fig. 6B). The overall pattern of changes was strongly suggestive for a lipid metabolic dysregulation in STEMI patients. Recently, whole blood choline (WBCHO) is used as an early marker for ACS patients, in addition to predicting cardiac ischemia in patients with negative troponin^40^.

One of the most intriguing findings in current results that indeed merit further investigation is H_2_S, which was significantly increased in the serum of STEMI patients (Figs 2B and 3B). H_2_S has been traditionally viewed as a toxic gas and less recognized as an endogenously generated biological mediator. It has recently been hypothesized that H_2_S is the “third endogenous signaling gasotransmitter” alongside with nitric oxide (NO) and carbon monoxide (CO)^41^. Endogenous H_2_S is generated in mammalian tissues by two pyridoxal-5′-phosphate-dependent enzymes, cystathionine-β-synthase (CBS) and cystathionine-γ–lyase (CSE)^42^. In the heart, endogenous H_2_S is synthesized from L-cysteine *via* CSE^43^. H_2_S can regulate heart contractility and protects the heart from ischemic injury^44^. The mechanism underlying the vascular relaxant effect of H_2_S are yet to be determined, although opening of ATP-sensitive K^+^ (K_ATP_) channels in vascular smooth muscle cells (SMCs) could mediate for such effect^45^. Recent studies^46^,^47^ showed an increase in CSE expression in the infarct area and area-at-risk beside the necrotic tissue. This demonstrates that H_2_S could be produced endogenously in myocardial tissue as a “compensatory response” to act as a cardioprotective agent against ischemic insult.

H_2_S was also found to promote angiogenesis and is anti-atherosclerotic in nature^48^,^49^. During atherosclerosis, there is an increase in the formation of reactive oxygen species (ROS). H_2_S can directly quench ROS with its strong reducing properties and inhibit ROS production^50^. Within the atherosclerotic lesion, there is a massive proliferation of vascular SMCs. H_2_S inhibits vascular SMCs proliferation and induces their apoptosis^51^. Foam cells formation from macrophages by oxidized LDL is critical for the initiation and progression of atherosclerotic lesions. H_2_S was shown to inhibit the formation of foam cells^52^.

There still remains some controversy over the cross talk between H_2_S and NO. Coletta *et al*.^53^ reported that the deficiency in endothelial nitric oxide synthase (eNOS) prevented the ability of H_2_S to induce angiogenesis, suggesting that NO is required for H_2_S to have its vascular effects. In a recent study^54^, exogenously administered H_2_S increased eNOS activity and NO bioavailability. Once we better understand how these molecules work together, we can begin building therapeutics that maximize the benefits of both signaling molecules.

In the current study, quantitative determination of H_2_S using ELISA showed that H_2_S was elevated in the serum of STEMI and UA patients, compared to normal levels in healthy controls (Fig. 5). However, distinct differences were observed in the level of H_2_S within ACS patients, with STEMI subjects showing the highest elevation. This leads to the obvious question whether the change in its level is correlated with the disease status. This targeted immunoassay confirmed the results obtained from the untargeted GC/MS based metabolomics approach (Figs 2B and 3B) and in accordance with recent findings showing that H_2_S levels are critically altered during myocardial ischemic injury^55^.

Therefore, elevation of H_2_S found in our study could be interpreted in different ways, with an initial explanation being a compensatory response to ischemia and endothelial dysfunction.

In conclusion, using such an untargeted metabolomics approach represents a paradigm shift in metabolic research, away from approaches which concentrated on single pathways (hypothesis-directed) to those which attempt to gain a comprehensive understanding of complex metabolic networks (hypothesis-generating)^38^. The current study uncovered a number of novel avenues for both identifying and diagnosing patients before they have major adverse cardiac events. That issue was addressed in the most challenging scenario, i.e., patients with spontaneous acute chest pain presenting at the triage, before admission to the ICU, and with normal baseline troponin levels. The metabolite biosignature presented herein shows high accuracy in discriminating STEMI patients from both healthy controls and UA patients. Nineteen marker metabolites were identified in the serum of STEMI patients, all of them are considered as potential biomarkers. These findings indicate that metabolite profiling techniques can develop a detailed picture of the metabolic changes that occur in response to the disease. Hence, they provide an opportunity to develop predictive biomarkers that will potentially allow for an earlier medical intervention.

Further studies are still needed to investigate the clinical implications of our findings. For example, UA patients before stabilization and AMI patients after stabilization should be investigated. Also the circadian variation in metabolites possibly interfering with their diagnostic power should be determined. Finally, interference with therapies should be investigated.

## Materials and Methods

### Ethics, consent and permissions

All procedures were designed according to the Declaration of Helsinki’s^56^. The study protocol was ethically reviewed and approved by the Ethics Review Committee of the German University in Cairo. Signed informed consent was obtained from all subjects prior to their inclusion in the study.

### Clinical characteristics of patients

Blood samples were collected from STEMI patients (n = 30), UA patients undergoing coronary angioplasty (n = 15), and sex- and age-matched healthy controls (n = 15) recruited from the National Heart Institute (NHI, Giza, Egypt).

In STEMI patients, samples were taken 1–2 h post chest pain, at the time of *triage*, and prior to the emergency department admission. Only patients diagnosed with a STEMI based on the admission ECG and/or elevated serial troponin levels were included in this study. It should be noted that samples were obtained when patients’ baseline troponin levels were normal and prior to the administration of any medications.

For the UA cohort, patients with a history of angina pectoris, ECG evidence of myocardial ischemia and coronary lesions suitable for angioplasty were recruited in this study. Two samples were obtained from each patient; one before the percutaneous coronary intervention, which is referred to as “before stent” and a second sample 3 h after the intervention, which is referred to as “after stent”.

Volunteers in the control group were included on the basis of a physician’s assessment of their general health status (body mass index, normal values in blood plasma and urine standard clinical tests, as well as the absence of major illness or chronic medication).

A detailed medical history, physical examination and biochemical profile were obtained for all subjects (Table 3). The exclusion criteria for both patients and controls included any concomitant acute or chronic severe diseases that would interfere with the evaluation of subjects (i.e., end-stage liver disease, hepatitis, hepatic insufficiency, pulmonary hypertension, renal failure, diabetes mellitus or any autoimmune disease). In addition, patients who had undergone any major surgical procedure within 14 days prior to the serum collection, or with a clinical history of MI, cardiomyopathy, congestive heart failure or depressed left ventricular function were also excluded from the study.

### Chemicals and reagents

N-methyl-N-(trimethylsilyl)-trifluoroacetamide (MSTFA) with 1% Trimethylsilyl chloride (TMCS), acetonitrile (99.8%), xylitol (an internal standard for relative quantification using GC/MS), pyridine, amino acids, sugars and standard *n*-alkanes mixture (C_8_-C_40_) were purchased from Sigma-Aldrich (St. Louis, Mo., USA). Water-*d*_2_ (99.80% d), and 2,2-dimethyl-2-silapentane-5-sulfonic acid (DSS) serving as an internal chemical shift NMR standard were provided from Deutero GmbH (Kastellaun, Germany).

### Sample collection

Five milliliters of blood were collected in sterile vaccutainers without an anticoagulant or preservative, then immediately stored at 4 °C to prevent sample degradation (<2 h). Later, samples were centrifuged (5810R, Eppendorf, Germany) at 4,000 rpm for 5 min and the resulting serum was aliquoted in batches of 500 μL and stored at −80 °C until analysis.

### Sample preparation for GC/MS and NMR analyses

For GC/MS analysis, 100 μL of serum was mixed with 5 μL xylitol (1 mg/mL, internal standard) and 200 μL of acetonitrile then centrifuged at 13,000 rpm for 10 min. The supernatant was dried using a speed vacuum concentrator (Eppendorf, Germany). For metabolites derivatization, 70 μL of MSTFA with 1% TMCS and 70 μL pyridine were added to the dried aliquot followed by incubation at 60 °C for 45 min.

For NMR analysis, 800 μL acetonitrile was added to 400 μL of thawed serum then centrifuged at 13,000 rpm for 10 min. The supernatant was carefully separated and dried. Dried aliquots were resuspended in 1.5 mL D_2_O containing 0.05% DSS, then the supernatant was transferred to a 5-mm NMR tube after centrifugation (13000 rpm for 5 min).

### GC/MS analysis

Analysis was performed on a Trace 1300 GC coupled to an ISQ LT– Single Quadrupole MSD (ThermoElectron, San Jose, USA) operating at conditions described previously by Farag *et al*.^57^. Chromatographic separation was achieved on a 30 m TG-5MS column (J&W Scientific; 0.32 mm ID, 1 μm film thickness, low polarity phase, chemically bonded with a 5% diphenyl and 95% dimethyl polysiloxane cross-linked stationary phase) at a constant flow of 0.5 mL min^−1^ with a temperature program of 80 °C for 2 min, ramped at 5 °C min^−1^ to 300 °C, and held for 5 min. To detect and eliminate retention time shifts, standard *n*-alkanes mixture (C_8_-C_40_) was injected into the GC/MS during analysis of each batch of samples.

### Identification of metabolites via GC/MS

Raw data acquired from Xcalibur 1.4 (Thermo Fisher Scientific, Inc., Waltham, MA) were exported in NetCDF format using the File Converter tool in Xcalibur software. An automated mass spectral deconvolution and identification system (AMDIS 2.64, NIST, Gaithersburg, Md., USA, www.amdis.net) was used to deconvolute the measured mass spectra prior to the database search. The RI was calculated relative to the standard *n*-alkanes mixture (C_8_-C_40_). The spectra of individual components were transferred to the NIST Mass Spectral Search Program MS Search 2.0. Identification of metabolites was performed by mass spectra matching against reference spectra of the NIST Mass Spectral Library 2005 (National Institute of Standardization and Technology, Gaithersburg, MD, USA), Golm Metabolome Database (Error! Hyperlink reference not valid. Golm.mpg.de/csbdb/gmd/home/gmd_sm.html) and Human Metabolome Database (HMDB, www.hmdb.ca/).

### GC/MS data processing for multivariate data analysis

XCMS data analysis software (http://www.137.131.20.83/download/) was used under R 2.9.2 environment^58^ for metabolite profiling using peak alignment, matching and identification, as described previously^57^,^59^. T2 and Distance to Model (DModX) tests were used to show whether the sample falls within a pre-defined range of variation or if the sample is an outlier. The quality of the OPLS model was assessed by the parameters R^2^ and Q^2^. R^2^ represents the goodness of fit, while Q^2^ represents the predictability of the model. The validity of the model was tested as described by Farag *et al*.^60^. The variables responsible for grouping of samples on the score plot were identified from the S loading plot of the OPLS-DA model. Multivariate data analysis was performed using the program SIMCA-P Version 13.0 (Umetrics, Umeå, Sweden).

An independent t test was performed using GraphPad Prism 5.0 software package (Version 5.01, San Diego, USA, www.graphpad.com) to investigate the levels of biomarker metabolites identified using PCA and OPLS-DA modeling at the univariate analysis level. A P value ≤ 0.05 was considered statistically significant.

### Headspace SPME-GC/MS analysis

SPME-GC/MS was used for analysis of serum volatile metabolites. A volume of 200 μL of serum was placed in 1.5 mL SPME vial. The vial was then sealed with a teflon lined magnetic cap using a hand crimper for volatiles collection. A 50 μm/30 μmDVB–CAR–PDMS metal SPME fiber (Supelco, USA) was inserted into the headspace above serum. The vial was placed at 50 °C and adsorption of volatiles was done for 30 min. Fibers were desorbed at 210 °C for 1 min in the injection port of a GC-17A gas chromatograph interfaced with a QP-5000 mass spectrometer (Shimadzu, Japan). GC separation of volatiles was carried out on a DB-5 ms column (Agilent, 30 m length, 0.25 mm inner diameter, and 0.25 μm film, non-polar phase, phenyl arylene polymer). Identification of volatiles was performed using the procedure described by Farag *et al*.^61^. Briefly, peaks were first deconvoluted using AMDIS software then identified by its RI relative to the standard *n*-alkanes mixture (C_8_-C_40_). Identities of metabolites were further confirmed by matching their mass spectra to NIST and WILEY library database.

### ^1^H-NMR analysis and quantification

All ^1^H-NMR spectra were recorded using an Agilent VNMRS 600 NMR spectrometer operating at a proton NMR frequency of 599.83 MHz and equipped with a 5-mm inverse detection cryoprobe. The parameters described by Farag *et al*.^59^ were used to record the ^1^H-NMR spectra. Briefly, scans were recorded with the following parameters: digital resolution 0.126 Hz/point, relaxation delay 23.7 s, pulse width (PW) 5.6 μs (90°), acquisition time 2.7 s and number of transient 160. Free induction decays were Fourier transformed with line broadening = 0.4 Hz. A combination of literature/database searches^9^,^62^, chemical shift, peak multiplicity and J coupling measurements were used for the assignment of NMR signals. Quantitative NMR analysis followed the exact procedure described by Farag *et al*.^57^ and without water suppression.

### ^1^H-NMR data processing for multivariate data analysis

ACD/NMR Manager lab version 10.0 software (Toronto, Canada) was used to automatically Fourier transform the NMR spectra to ESP files. The singlet produced by DSS methyl groups was used as an internal standard for chemical shift referencing (set to 0 ppm). The spectra were then divided within the region of δ −0.4–9 ppm into evenly spaced windows, named bins or buckets, whose width = 0.04 ppm. The regions of residual water (δ 4.7–4.9) and acetonitrile signals (δ 3.33–3.39) were removed prior to the multivariate data analyses. PCA was performed using R package (2.9.2) by employing custom-written scripts after exclusion of solvent regions and scaling to DSS signal. T2 and Distance to Model (DModX) tests were used to show whether the sample falls within a pre-defined range of variation or if the sample is an outlier. The quality of the OPLS model was assessed by the parameters R^2^ and Q^2^. R^2^ represents the goodness of fit, while Q^2^ represents the predictability of the model. The validity of the model was tested as described by Farag *et al*.^60^. The variables responsible for grouping of samples on the score plot were identified from the S loading plot of the OPLS-DA model.

Multivariate data analysis was performed using the program SIMCA-P Version 13.0 (Umetrics, Umeå, Sweden).

An independent t test was performed using GraphPad Prism 5.0 software package (Version 5.01, San Diego, USA, www.graphpad.com) to investigate the levels of biomarker metabolites identified using PCA and OPLS-DA modeling at the univariate analysis level. A P value ≤ 0.05 was considered statistically significant.

### Metabolic pathway analysis

The identified marker metabolites were mapped through their respective metabolic pathways using Kyoto Encyclopedia of Genes and Genomes (KEGG) pathway database (http://www.genome.jp/kegg/pathway.html) and an interactive metabolic pathways map (Sigma-Aldrich, USA, http://www.sigmaaldrich.com/technical-documents/articles/biology/interactive metabolic-pathways-map.html).

### Quantitative determination of serum H_2_S using ELISA

The human H_2_S kit (New Test Co, USA) uses a quantitative double-antibody sandwich ELISA to assay the level of serum H_2_S in samples. Standard solutions over a range of concentrations were prepared *via* serial dilution of the standard stock solution (3200 pg/mL). For the standard wells, 50 uL of standard solution and 50 uL of streptavidin-HRP were added to the appropriate well in the antibody pre-coated microtiter plate. For the sample wells, 40 uL of the serum to be tested, 10 uL of H_2_S-antibody and 50 uL of streptavidin-HRP were added to each well. The plate was covered and incubated at 37 °C for 60 min. After the incubation period, wells were decanted and washed five times with a 30× wash solution. The wells were then incubated in dark with 50 uL chromogen A and 50 uL chromogen B at 37 °C for 10 min. To stop the reaction, 50 uL of the stop solution was added to each well which turned the color of the solution into yellow immediately. For the blank wells, only chromogen A, B and the stop solution were added. Duplicates were carried out for standard, sample and blank wells. The optical density (O.D.) was determined spectrophotometrically at 450 nm using a microplate reader (Victor^3^ V, USA) and the average of duplicate readings was calculated for all wells. A calibration curve was plotted relating the concentration of each standard solution on the horizontal (X) axis to the corresponding average O.D. on the vertical (Y) axis. The standard curve linear regression equation was calculated and serum H_2_S concentration in each sample was interpolated from this standard curve.

One-way analysis of variance (ANOVA) and tukey multiple comparison test were employed using GraphPad Prism 5.0 software package (Version 5.01, San Diego, USA, www.graphpad.com). Data are represented as mean ± SEM. A P value ≤ 0.05 was considered statistically significant.

## Additional Information

**How to cite this article**: Ali, S. E. *et al*. A Comparative Metabolomics Approach Reveals Early Biomarkers for Metabolic Response to Acute Myocardial Infarction. *Sci. Rep.* **6**, 36359; doi: 10.1038/srep36359 (2016).

**Publisher’s note:** Springer Nature remains neutral with regard to jurisdictional claims in published maps and institutional affiliations.

## Supplementary Material

Supplementary Information

## Figures and Tables

**Figure 7:**
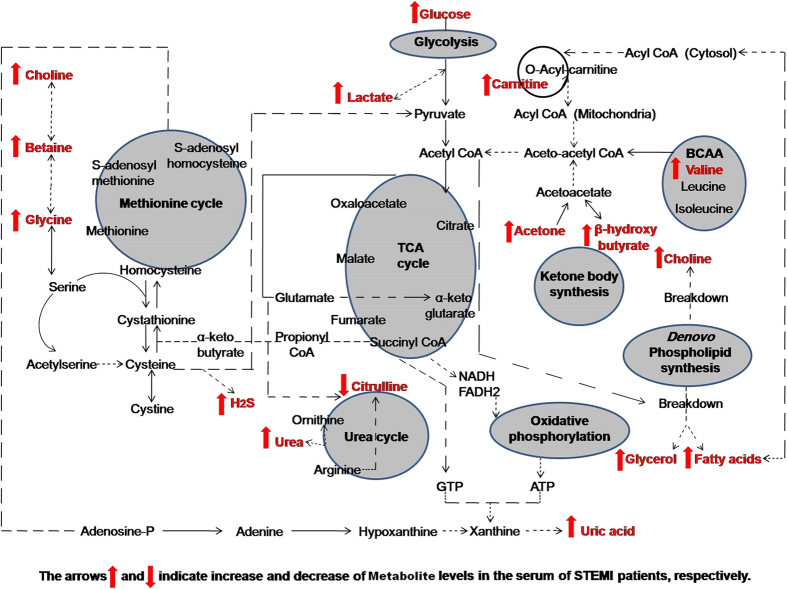
A detailed map illustrating the most predominant altered metabolic pathways and the biochemical linkages among the biomarker metabolites identified in the serum of STEMI patients.

**Table 1:** Marker metabolites identified in PCA and OPLS-DA models of GC/MS based metabolite profiling.

Peak no.	rt (min)	*m/z*	P value ^a^	Metabolites	Variations versus healthy controls ^b^
M3	7.612	171	0.0197	H_2_S	↑
M8	10.317	117	<0.0001	β-Hydroxybutyric acid	↑
M9	10.358	191	0.0014	Lactic acid	↑
M18	12.306	131	0.0042	α-Hydroxyisobutyric acid	↑
M22	15.377	189	0.0071	Urea	↑
M25	16.54	205	0.0126	Glycerol	↑
M41	24.553	186	0.0064	L-Valine	↑
M48M49	29.07729.077	156155	0.00060.0018	Citrulline	↓↓
M57M65	31.96733.581	204204	0.0035<0.0001	Glucose	↑↑
M67	34.38	313	0.0011	Palmitic acid	↑
M69	35.865	441	<0.0001	Uric acid	↑
M72	37.526	337	0.0024	Linoleic acid	↑
M73	37.6	339	0.0326	Oleic acid	↑
M74	37.967	341	0.0236	Stearic acid	↑

^a^The P value was calculated from independent samples t test.

^b^The arrows ↑ and ↓ indicate increase and decrease of metabolite levels in the serum of STEMI patients as compared to healthy controls, respectively.

**Table 2:** Marker metabolites identified in PCA and OPLS-DA models of ^1^H-NMR based metabolite fingerprinting.

Bin no.	Chemical shift (ppm)	P value^a^	Metabolites	Variations versus healthy controls^b^
44	1.3200–1.3600	0.0215	Lactic acid	↑
111	4.0702–4.1102	0.0236		↑
92	3.2400–3.2800	<0.0001	Carnitine	↑
92	3.2400–3.2800	<0.0001	Betaine	↑
92	3.2400–3.2800	<0.0001	D-glucose	↑
94	3.3902–3.4302	<0.0001		↑
95	3.4302–3.4702	0.0016		↑
96	3.4702–3.5102	<0.0001		↑
97	3.5102–3.5502	<0.0001		↑
102	3.7102–3.7502	0.0207		↑
103	3.7502–3.7902	0.0024		↑
105	3.8302–3.8702	0.0010		↑
106	3.8702–3.9102	0.0116		↑
96	3.4702–3.5102	<0.0001	Choline	↑
97	3.5102–3.5502	<0.0001	Glycerol	↑
97	3.5102–3.5502	<0.0001	Glycine	↑
112	4.1102–4.1502	0.0360	β-Hydroxybutyric acid	↑
125	4.6302–4.6702	0.0001	β -glucose	↑
135	5.2202–5.2602	<0.0001	α-glucose	↑

^a^The P value was calculated from independent samples t test.

^b^The arrows ↑ and ↓ indicate increase and decrease of metabolite levels in the serum of STEMI patients as compared to healthy controls, respectively.

**Table 3:** Baseline characteristics of participants involved in this study.

	STEMI patients (n = 30)	UA patients (Before stent; n = 15) (After stent; n = 15)	Healthy controls (n = 15)
Age ± SD (years)	54 ± 14.5	58 ± 11	57 ± 13.2
Sex (male/female)	24/6	8/7	7/8
Smokers	16	11	5
Ex-smokers	1	1	3
Blood pressure	136/87 ± 7	135/82 ± 3	125/85 ± 5.5
Random blood glucose (mg/dL)	168 ± 6.4^*^	104 ± 5.2	96.8 ± 8.7
Total cholesterol (mg/dL)	207.6 ± 7.2	211.6 ± 9.07	199 ± 5.75
Triglycerides (mg/dL)	153 ± 8.3	151 ± 10	118.9 ± 4.8
Serum creatinine (mg/dL)	0.95 ± 0.2	0.85 ± 0.2	0.9 ± 0.2
**ECG**	ST segment elevation	No ST segment elevation	Normal
hs-CRP (mg/dL)	3.8 ± 1.23^*^	1.3 ± 0.47	0.8 ± 0.25
Serum troponin (1–2 h post chest pain) (ng/mL)	0.02 ± 0.01	0.02 ± 0.01	0.02 ± 0.01
Serial serum troponin (6–8 h post chest pain) (ng/mL)	5.36 ± 1.5^*^	0.02 ± 0.01	0.02 ± 0.01

Data are represented as mean ± SEM. A P value ≤ 0.05 was considered statistically significant.
