# Supplementary material for: A Comparative Metabolomics Approach Reveals Early Biomarkers for Metabolic Response to Acute Myocardial Infarction
Source: Sci Rep. 2016 Nov 8;6:36359. doi: 10.1038/srep36359 (PMC5099572; doi:10.1038/srep36359)

SUPPLEMENTARY INFORMATION

A Comparative Metabolomics Approach Reveals Early Biomarkers for Metabolic Response to Acute Myocardial Infarction

Sara E. Ali [1], Mohamed A. Farag [2*], Paul Holvoet [3], Rasha S. Hanafi [4], Mohamed Z. Gad [5]

[1] Department of Pharmaceutical Biology, Faculty of Pharmacy & Biotechnology, The German University in Cairo, Egypt

[2] Department of Pharmacognosy, Faculty of Pharmacy, Cairo University, Cairo, 11562, Egypt

[3] Department of Cardiovascular Sciences, Atherosclerosis and Metabolism Unit, Katholieke Universiteit Leuven, Belgium

[4] Department of Pharmaceutical Chemistry, Faculty of Pharmacy & Biotechnology, The German University in Cairo, Egypt

[5] Department of Biochemistry, Faculty of Pharmacy & Biotechnology, The German University in Cairo, Egypt

*Corresponding author: Mohamed.farag@pharma.cu.edu.eg, mfarag73@yahoo.com, Tel: +20-1004142567

Table of contents

Supplementary Tables

Page	Supplementary Table	Description
**S-3**	**S1**	Summary of GC/MS analysis identified metabolites in serum samples from STEMI patients, UA patients and healthy controls.
**S-11**	**S2**	Summary of ^{1}H-NMR analysis identified metabolites in serum samples from STEMI patients, UA patients and healthy controls.

Supplementary Figures

Page	Supplementary Figure	Description
**S-6**	**S1**	GC/MS based OPLS-DA of STEMI patients (▲) versus healthy controls (●) after removing major peaks revealed from the first OPLS analysis.
**S-7**	**S2**	GC/MS based PCA of the fatty acids profile of STEMI patients (▲), before stent samples of unstable angina patients (▽) and healthy controls (●).
**S-8**	**S3**	GC/MS based OPLS-DA of the fatty acids profile of STEMI patients (▲), before stent samples of unstable angina patients (▽) and healthy controls (●).
**S-9**	**S4**	SPME-GC/MS of a healthy control (■) and a STEMI patient (■).
**S-10**	**S5**	A 600 MHz ^{1}H-NMR spectrum of a healthy human serum with expanded spectral region (B and C).
**S-14**	**S6**	^{1}H-NMR based PCA of STEMI patients (▲) versus healthy controls (●).
**S-15**	**S7**	^{1}H-NMR quantification of target compounds in serum of STEMI patients versus healthy controls.
**S-16**	**S8**	NMR based OPLS-DA of STEMI patients (▲) versus healthy controls (●).

Table S1

Summary of GC/MS analysis identified metabolites in serum samples from STEMI patients, UA patients and healthy controls.

Peak	Metabolite	rt (min)	*m/z*	RI	RI Golm library
**M1**	unknown	6.107	147	912	
**M2**	Ethanol,2-(methylamino)	7.136	128	948	
**M3**	Hydrogen sulfide (2 TMS)	7.612	171	964.9	955.7
**M4**	Pentanoic acid (1 TMS)	8.076	159	981	
**M5**	unknown	8.252	191	984	
**M6**	β-Hydroxybutyric acid (2 TMS)	8.67	117	1011	
**M7**	Hydrocarbon	9.701	136	1037.9	1036.9
**M8**	β-Hydroxybutyric acid (2 TMS)	10.317	117	1054	
**M9**	Lactic acid (2 TMS)	10.358	191	1060.9	1044.7
**M10**	2-Aminobutyric acid (2 TMS)	10.525	130	1065	
**M11**	Hexanoic acid (1 TMS)	10.636	173	1070.7	1062
**M12**	Acetic acid (2 TMS)	10.757	205	1075	
**M13**	Pyruvic acid (2 TMS)	11.123	217	1087.6	1081.7
**M14**	L-Valine (1 TMS)	11.19	174	1099	
**M15**	Alanine (2 TMS)	11.591	190	1104	1087.4
**M16**	Malonic acid (2 TMS)	11.632	204	1107	
**M17**	2-Ketoisocaproic acid (1 TMS)	11.811	143	1112	
**M18**	α-Hyroxyisobutyric acid (2 TMS)	12.306	131	1128.6	1112.5
**M19**	β-Hydroxybutyric acid (2TMS)	13.235	191	1160.5	1148.4
**M20**	α-Hydroxyvaleric acid (2 TMS)	13.435	219	1165	
**M21**	L-Valine (2 TMS)	14.909	218	1220	1207.1
**M22**	Urea (2 TMS)	15.377	189	1240.1	1235.1
**M23**	Benzoic acid (1 TMS)	15.85	179	1251.4	1251.3
**M24**	Serine (2 TMS)	16.116	132	1260.5	1254
**M25**	Glycerol (3 TMS)	16.54	205	1275.8	1262.2
**M26**	L-Isoleucine (2 TMS)	17.182	158	1298.8	1286.7
**M27**	L-Proline (2 TMS)	17.334	142	1304.4	1295.7
**M28**	Glycine (3 TMS)	17.575	174	1313.8	1302.1
**M29**	Tridecane	17.901	112	1325.4	1293.4
**M30**	Glyceric acid (3 TMS)	18.19	189	1336.1	1320.3
**M31**	L-Serine (3 TMS)	19.019	204	1366.6	1351.3
**M32**	L-Threonine (3 TMS)	19.768	218	1394.4	1377.2
**M33**	Aminomalonic acid (3TMS)	21.955	218	1479	
**M34**	Aspartic acid (3 TMS)	22.06	232	1483	
**M35**	L-Norvaline (2 TMS)	22.405	188	1497	
**M36**	Erthyritol (4 TMS)	22.973	217	1520	1493.3

**M37**	Pyroglutamic acid (2 TMS)	23.29	156	1532.9	1521.7
**M38**	Erythronic acid (4 TMS)	23.875	292	1556.9	1529
**M39**	α-Aminoadipic acid (3 TMS)	24.048	260	1564	
**M40**	Creatinine (3 TMS)	24.276	115	1572.2	1553.7
**M41**	L-Valine (2 TMS)	24.553	186	1585	
**M42**	L-Proline (2 TMS)	24.727	216	1591.8	1583.1
**M43**	Glutamine (3 TMS)	25.318	246	1617	
**M44**	L-Phenylalanine (2 TMS)	25.877	218	1641.1	1629.6
**M45**	Dodecanoic acid (1 TMS)	26.117	257	1651.5	1654.1
**M46**	*N*-acetyl glycine (2 TMS)	26.79	288	1681	1660.3
**M47**	Xylitol (5TMS)	27.89	217	1730	1694.6
**M48**	Citrulline (4 TMS)	29.077	156	1784	
**M49**	Citrulline (4 TMS)	29.077	155	1784	
**M50**	D-Mannose (5 TMS)	30.089	204	1831	
**M51**	D-Fructose (5 TMS)	30.197	217	1836.4	1789.8
**M52**	Citric acid (4 TMS)	30.21	273	1839	
**M53**	D-Fructose (5 TMS)	30.424	204	1847	
**M54**	Myristic acid (1 TMS)	30.457	285	1849	
**M55**	Myo-Inositol (6 TMS)	31.4	217	1913	
**M56**	Mannose (5 TMS)	31.895	147	1918.5	1880
**M57**	Glucopyranose (5 TMS)	31.967	204	1922	1879.9
**M58**	D-Galactose (5 TMS)	32.141	217	1931	
**M59**	D-Mannose (5 TMS)	32.213	204	1934.5	1959.2
**M60**	Lysine (4 TMS)	32.256	174	1936.5	1913.6
**M61**	Histidine (3 TMS)	32.322	154	1939.8	1914.5
**M62**	Pentanoic acid (1 TMS)	32.431	299	1946.4	1945.5
**M63**	L-Tyrosine (3 TMS)	32.636	218	1955.6	1934.1
**M64**	Glucitol (6 TMS)	32.768	319	1962	
**M65**	α-D-Glucopyranose (5 TMS)	33.581	204	2003.1	1959.2
**M66**	Palmitelaidic acid (1 TMS)	33.942	311	2022	
**M67**	Palmitic acid (1 TMS)	34.38	313	2045	
**M68**	*N*-acetylglutamine (4 TMS)	35.348	203	2096	
**M69**	Uric acid (4 TMS)	35.865	441	2124.6	2094.6
**M70**	Myo-inositol (6 TMS)	35.947	318	2128.8	2080.3
**M71**	Heptadecanoic acid (1 TMS)	36.226	327	2144.4	2144.6
**M72**	Linoleic acid (1 TMS)	37.526	337	2215.9	2219.1
**M73**	Oleic acid (1 TMS)	37.6	339	2220	2216.6
**M74**	Stearic acid (1 TMS)	37.967	341	2240.8	2243.5
**M75**	L-Tryptophan (3 TMS)	38.101	202	2247.5	2217.5
**M76**	Pseudo uridine (5 TMS)	40.052	217	2355	
**M77**	Arachidonic acid (1 TMS)	40.3	129	2368.4	2383.7
**M78**	Oleamide (1 TMS)	40.981	338	2400	

**M79**	Arachidic acid (1 TMS)	41.316	369	2424.3	2453.8
**M80**	Monopalmitoyl glycerol (2 TMS)	43.191	218	2527	
**M81**	Docosahexanoic acid (1 TMS)	43.37	159	2537.3	2575.9
**M82**	Monopalmitoyl glycerol isomer (2 TMS)	43.716	371	2556	
**M83**	Cholesterol (1 TMS)	43.868	368	2564.5	3188.2
**M84**	1-Monooleoylglycerol (2 TMS)	46.286	397	2688	
**M85**	Monostearin (2 TMS)	46.572	399	2713	

Figure S1

GC/MS based OPLS-DA of STEMI patients (▲) versus healthy controls (●) after removing major peaks revealed from the first OPLS analysis. (A) OPLS-DA score plot (B) loading S-plot derived from samples of the two groups modeled against each other. The S-plot shows the covariance p[1] against the correlation p(cor)[1] of the variables of the discriminating component of the OPLS-DA model. Selected variables are highlighted in the S-plot with each metabolite denoted by its mass/RT (min) value: M18; α-hydroxyisobutyric acid, M41; valine, M48&49; citrulline, M67; palmitic, M69; uric acid. Peak numbers correspond to those listed in (**Supplementary Table S1**).

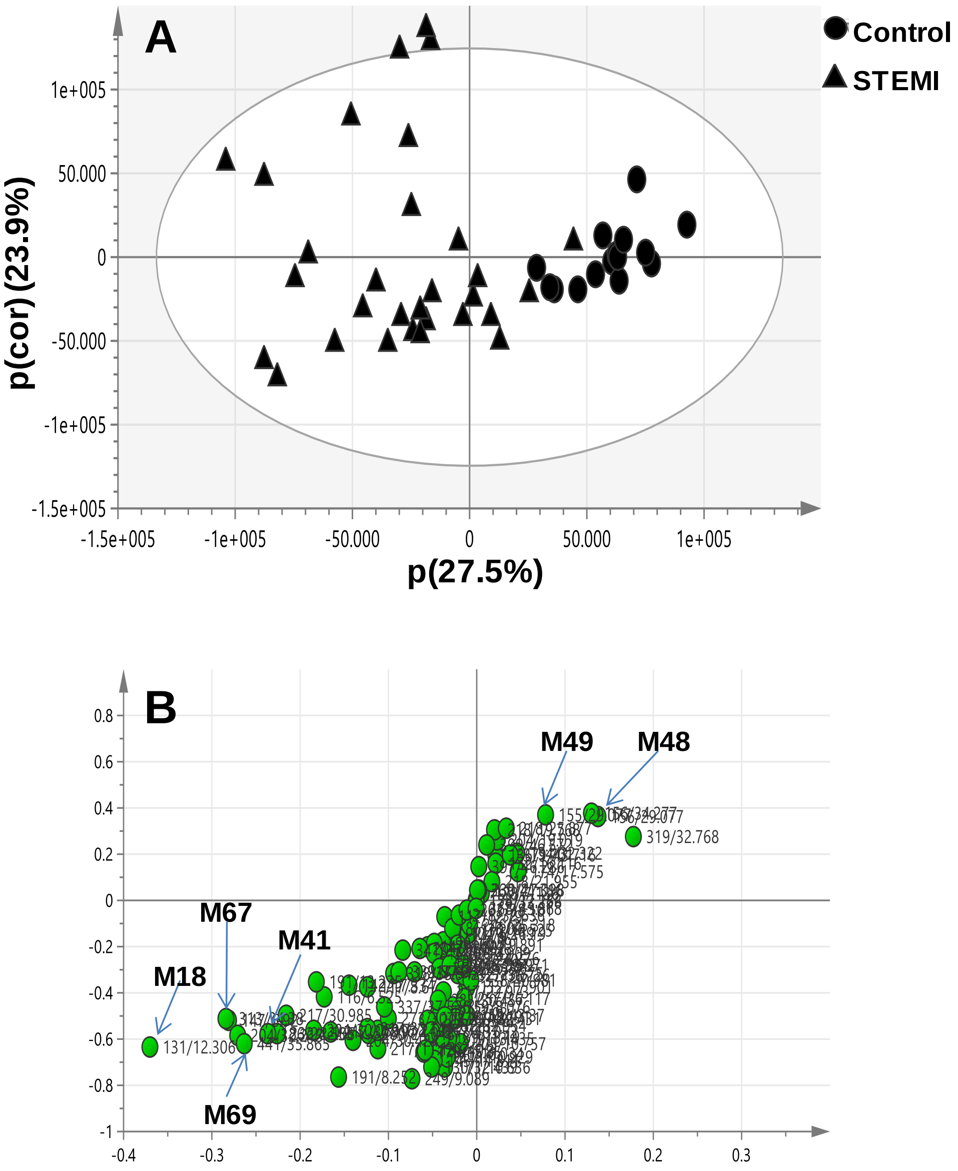

Figure S2

GC/MS based PCA of the fatty acids profile of STEMI patients ( ), before stent samples of unstable angina patients ( ) and healthy controls ( ). (A) Score plot of PC1 and PC2 scores (B) Loading plot for PC1 components contributing peaks and their assignments, with each metabolite denoted by its mass/rt (min) value: M67; palmitic acid, M72; linoleic acid, M73; oleic acid, M74; stearic acid. Peak numbers correspond to those listed in (**Supplementary Table S1**).

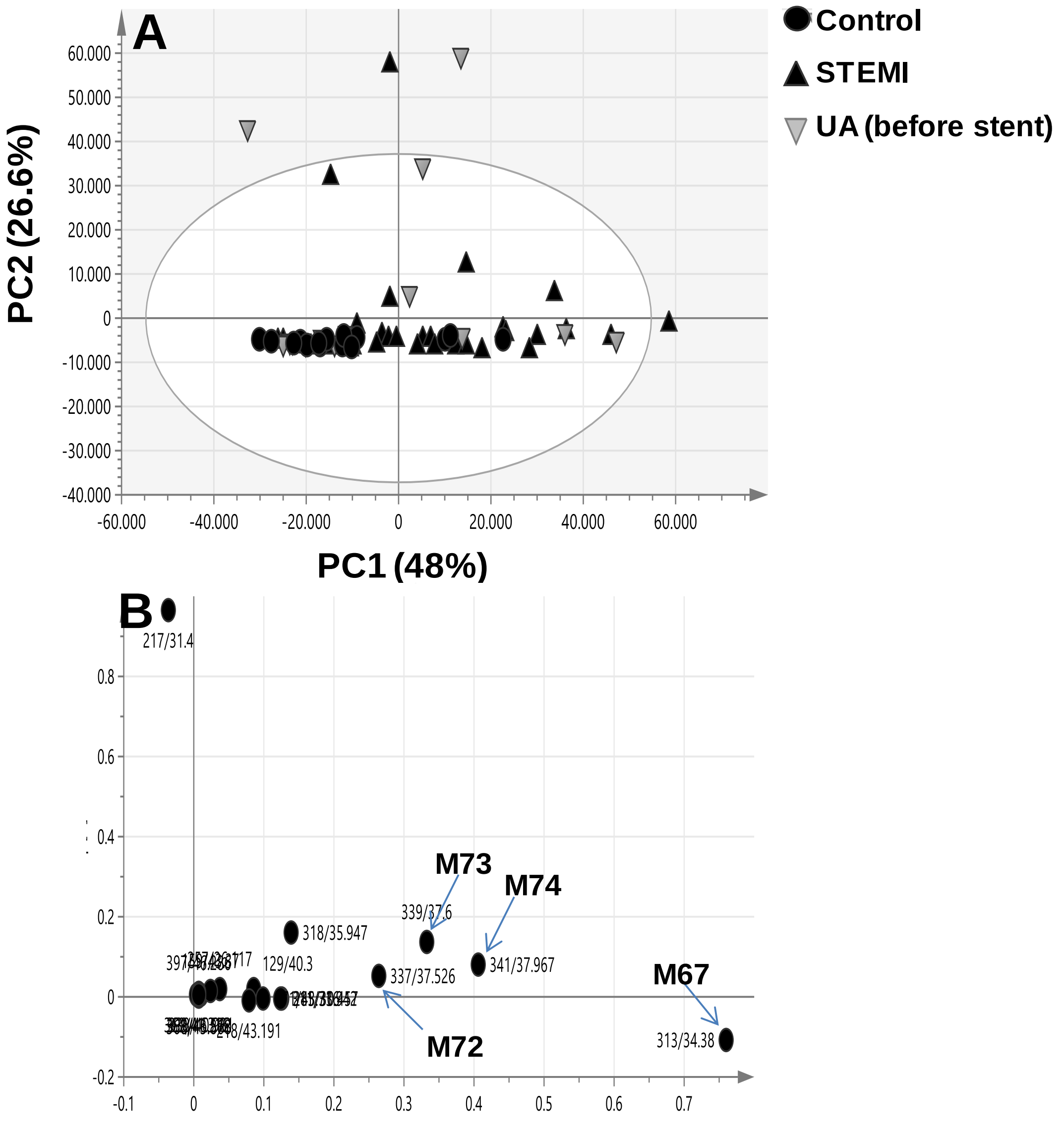

Figure S3

GC/MS based OPLS-DA of the fatty acids profile of STEMI patients (▲), before stent samples of unstable angina patients (▽) and healthy controls (●). (A) OPLS-DA score plot (B) loading plot derived from samples modeled against each other. The loading plot shows the covariance p[1] against the correlation p(cor)[1] of the variables of the discriminating component of the OPLS-DA model. Selected variables are highlighted in the S-plot with each metabolite denoted by its mass/RT (min) value: M67; palmitic acid. Peak numbers correspond to those listed in (**Supplementary Table S1**).

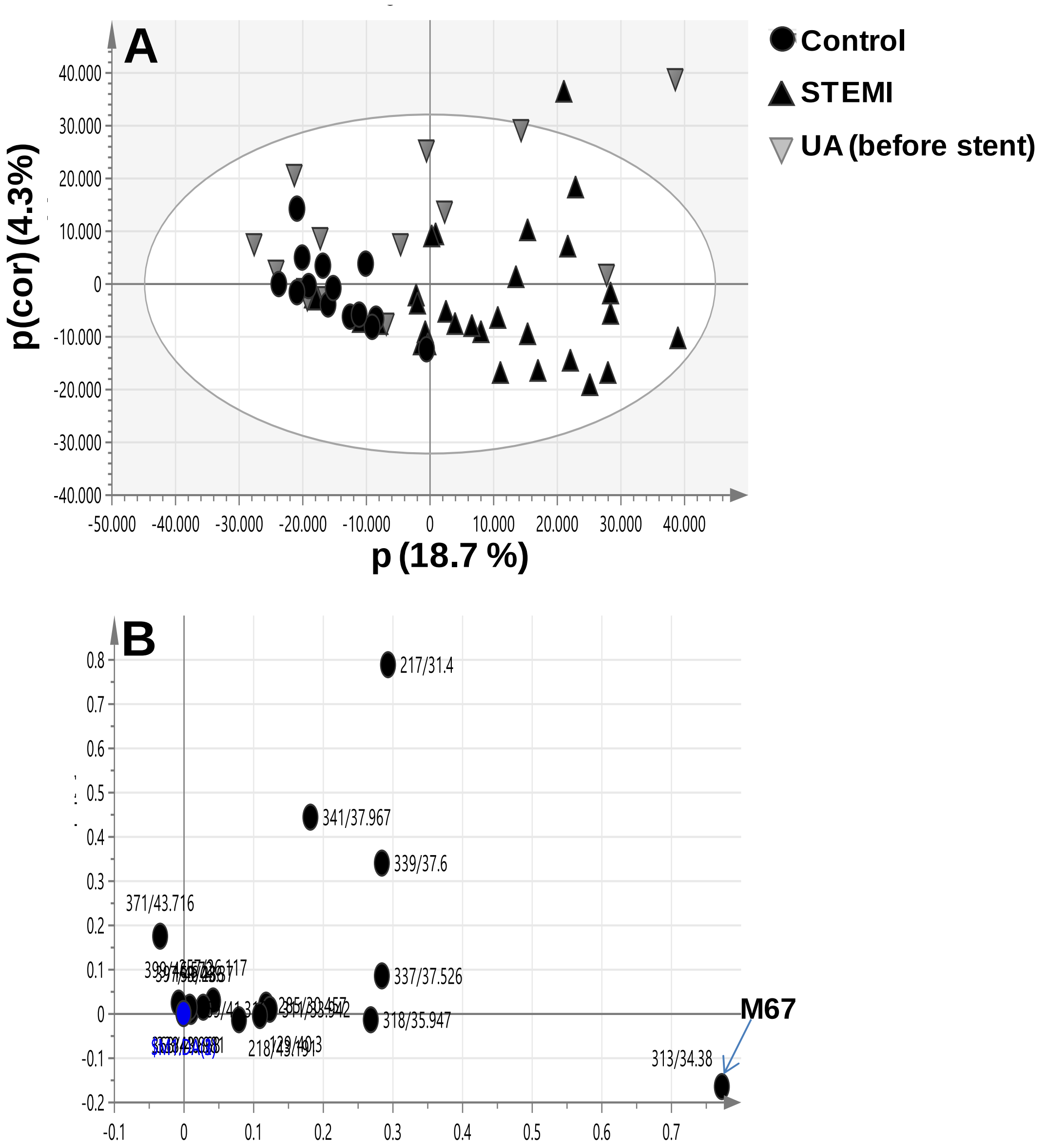

Figure S4

SPME-GC/MS of a healthy control ( ) and a STEMI patient ( ).

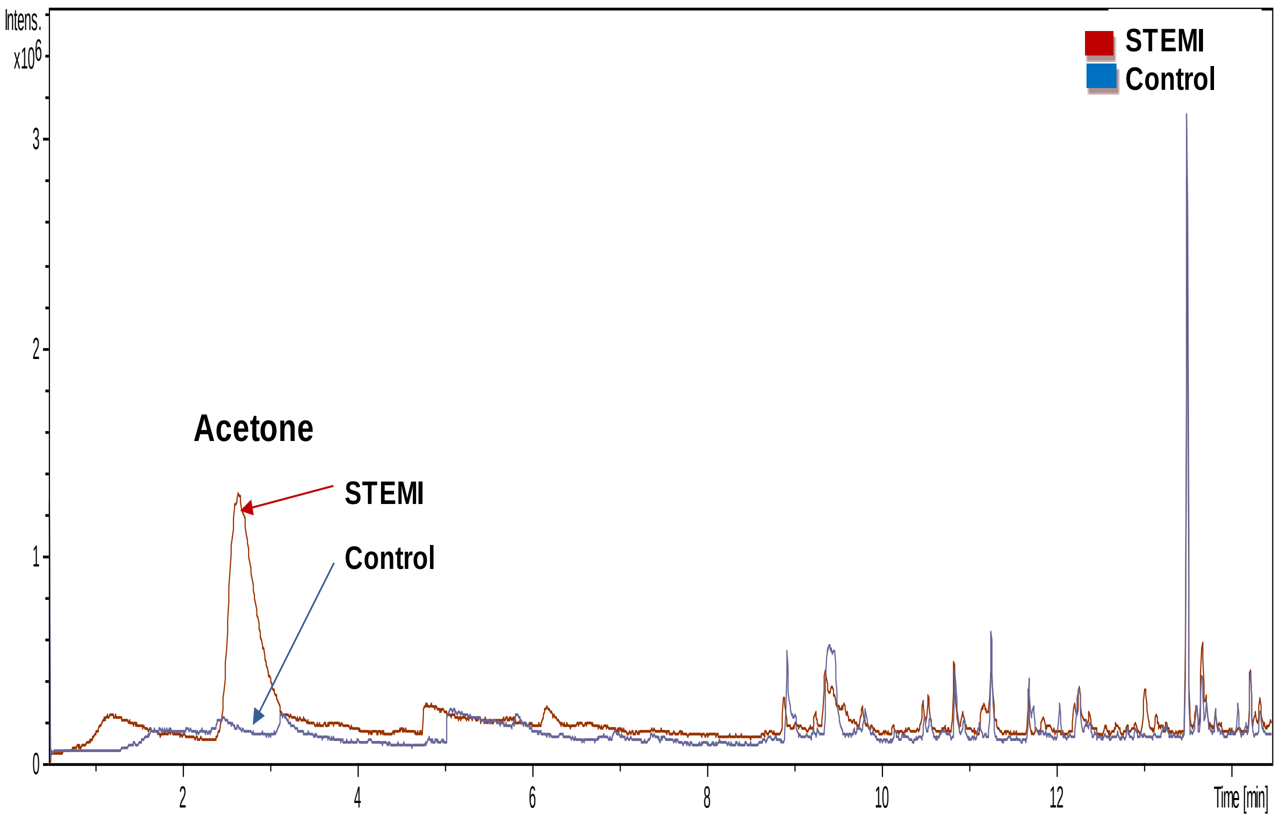

Figure S5

A 600 MHz ^{1}H-NMR spectrum of a healthy human serum with expanded spectral region (B and C).

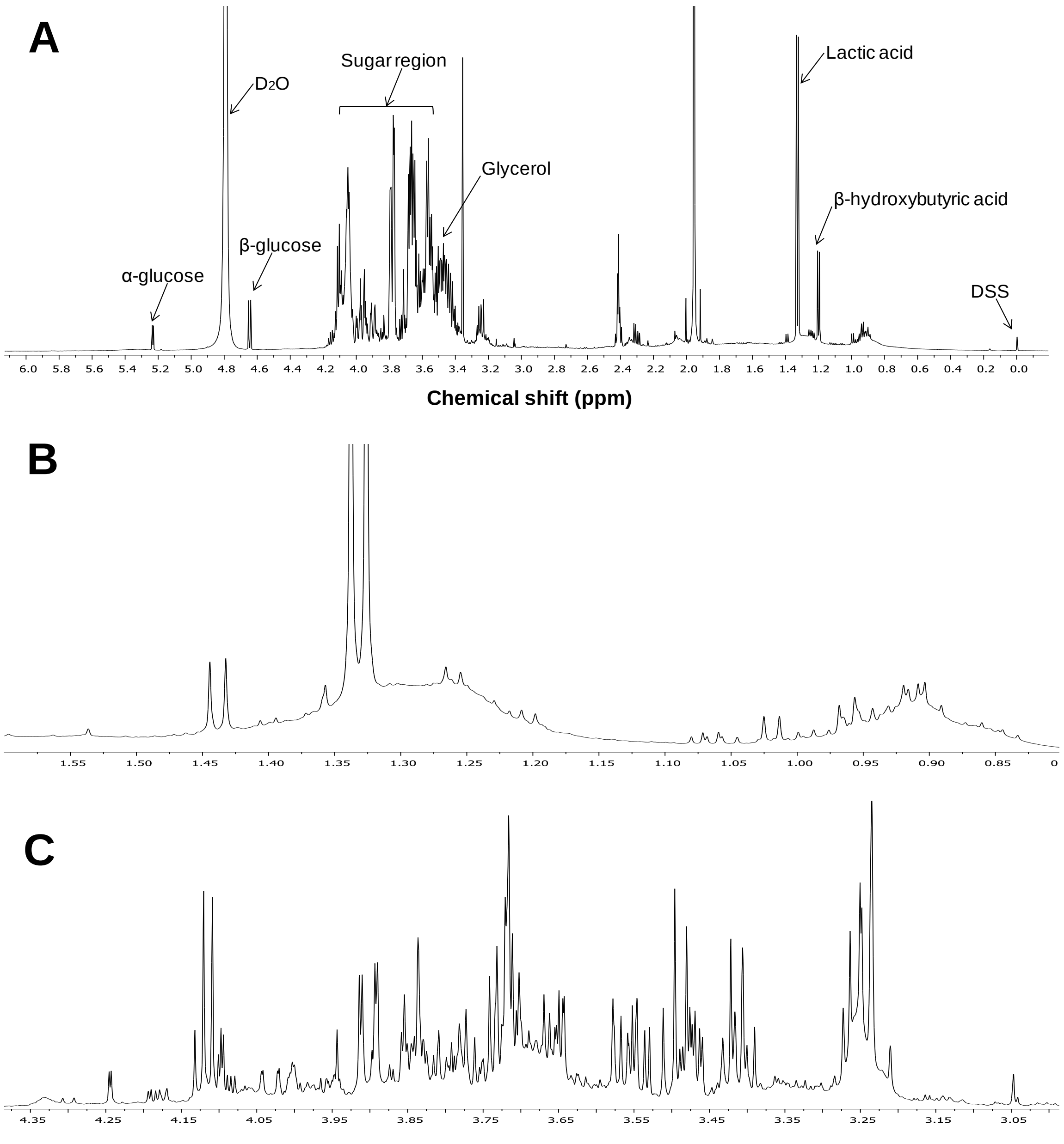

Table S2

Summary of ^{1}H-NMR analysis identified metabolites in serum samples from STEMI patients, UA patients and healthy controls.

Number	Metabolite	^{1}H shift (ppm)	Multiplicity	Coupling constant (Hz)
**N1**	1-Methylhistidine	7.114	s	
		7.927	s	
**N2**	1,2-Propanediol	1.144	d	J=6.5
		3.441	dd	J=11.5, 4.4
		3.882	m	
**N3**	2-Hydroxyisovaleric acid	2.017	m	
**N4**	2-Oxoisocaproic acid	0.934	d	J=6.5
		2.085	m	
		2.612	d	J=7.0
**N5**	2-Oxoisovaleric acid	1.126	d	J=7.0
**N6**	β-Hydroxybutyric acid	1.200	d	J=6.3
		2.305	m	
		2.414	m	
		4.156	m	
**N7**	3-Hydroxyisovaleric acid	1.269	s	
**N8**	Acetic acid	1.916	s	
**N9**	Acetone	2.233	s	
**N10**	Acetylcarnitine	3.194	s	
**N11**	N-Acetylglycine	2.054	s	
**N12**	Alanine	1.433	d	J=6.9
**N13**	Arginine	1.664	m	
		3.226	t	J=6.1
**N14**	Arginine/Lysine*	1.722	m	
		1.734	m	
		1.909	m	
**N15**	Asparagine	2.972	d	J=3.4
		2.929	d	J=3.5
**N16**	Aspartic acid	3.900	dd	J= 14.4, 2.3
**N17**	Betaine	3.268	s	
**N18**	Carnitine	3.235	s	
**N19**	Choline	3.200	s	
		3.506	dd	J=5.9, 3.5
		4.052	m	
**N20**	Citric acid	2.545	d	J=15.1
**N21**	Creatine	3.038	s	
		3.933	s	
**N22**	Creatinine	3.044	s	
		4.050	s	
**N23**	Dimethylglycine	2.942	s	
**N24**	Dimethylamine/Sarcosine*	2.730	s	
**N25**	Ethanolamine	3.131	d	J=5.8

**N26**	α-Glucose	3.842	m	
		5.232	d	J=3.8
**N27**	β-Glucose	3.244	dd	J=9.3, 8.0
		3.900	dd	J=12.3, 1.9
		4.645	d	J=8.0
**N28**	α/β-Glucose*	3.405	m	
		3.458	m	
		3.519	dd	J=10.3, 3.8
		3.458	m	
		3.526	dd	J=9.5, 3.6
		3.715	m	
		3.728	m	
		3.829	m	
**N29**	Glutamic acid	2.041	m	
		2.118	m	
		2.349	m	
		2.355	m	
		3.746	dd	J=7.0, 5.1
**N30**	Glutamine	2.131	m	
		2.452	m	
**N31**	Glycerol	3.550	d	J=5.9
		3.569	d	J=5.9
		3.636	d	J=4.4
**N32**	Glycine	3.548	s	
**N33**	Histidine	7.205	s	
		7.090	d	J=0.6
		8.101	s	
**N34**	Isoleucine	0.966	t	J=7.2
		3.671	d	J=3.5
		3.661	d	J=4.5
**N35**	Isoleucine/Glycerol*	3.680	d	J=4.4
**N36**	Lactic acid	1.328	d	J=6.9
		4.109	dd	J=6.9, 7.0
**N37**	Lactic acid/Proline/Pyroglutamic acid*	4.151	dd	J=13.4, 6.4
**N38**	Leucine	1.698	m	
**N39**	Lysine	1.435	m	
**N40**	α-Mannose	5.181	d	J=1.5
**N41**	β-Mannose	4.900	d	J=0.9
**N42**	Methionine	3.851	dd	J=7.4, 5.2
**N43**	N-Methyl-α-aminoisobutyric acid	1.597	s	
**N44**	Proline	1.949	m	
		2.057	m	
**N45**	Pyruvic acid	2.509	s	
		2.479	s	
		2.468	s	
		2.454	s	
		2.442	s	
**N46**	Serine	3.998	d	J=3.3
		3.944	d	J=3.8

		4.021	d	J=3.1
**N47**	Succinic acid	2.410	s	
**N48**	Sucrose	5.421	d	J=3.7
**N49**	Threonine	1.303	d	J=6.3
		1.391	d	J=7.2
		3.592	d	J=4.4
		4.244	m	
**N50**	Tryptophan	7.329	s	
		7.546	d	J=0.7
		7.741	d	J=7.9
**N51**	Tyrosine	7.206	d	J=8.6
		7.179	d	J=8.5
**N52**	Urea	5.784	s	
**N53**	Uridine	5.923	d	J=4.8
**N54**	Valine	0.994	d	J=7.0
**N55**	Xanthine	8.032	s	

*** Corresponds to overlapping NMR signals**

Figure S6

^{1}H-NMR based PCA of STEMI patients (▲) versus healthy controls (●). (A) Score plot of PC1 and PC2 scores (B) Loading plot for PC2 components contributing bin numbers. Signals were assigned in each bin as follows: Bin 44, lactic acid ; bin 94, D-glucose; bin 96, choline and D-glucose; bin 97, glycerol, glycine and D-glucose; bin 105, D-glucose; bin 111, lactic acid; bin 112, β-hydroxybutyric acid ; bin 125, β-glucose.

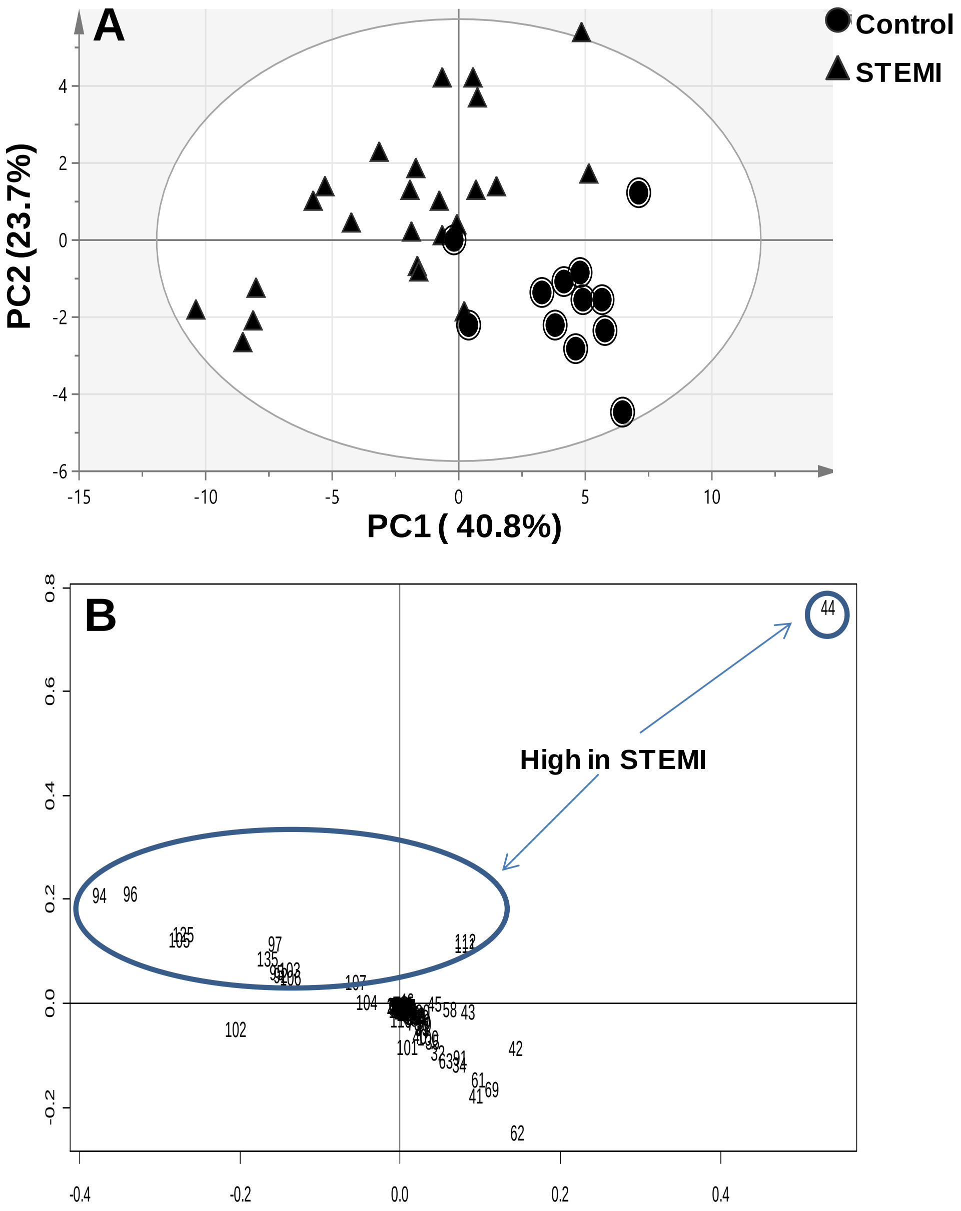

Figure S7

^{1}H-NMR quantification of target compounds in serum of STEMI patients versus healthy controls. NMR signals (1.33, d, J=6.9) for lactic acid; (5.23, d, J=3.8) for α-glucose and (4.65, d, J=8.0) for β-glucose were used for quantification. Results are expressed as mean ± SEM. A p value ≤ 0.05 was considered statistically significant.

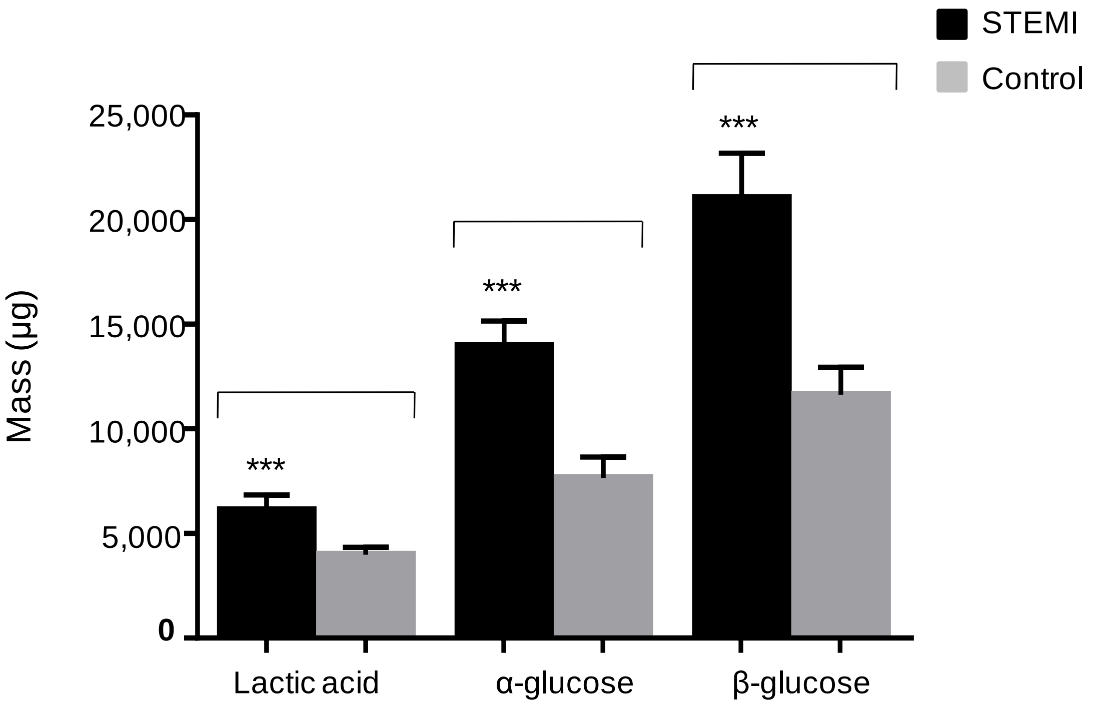

Figure S8

NMR based OPLS-DA of STEMI patients (▲) versus healthy controls (●). (A) OPLS-DA score plot (B) loading plot derived from samples modeled against each other. The loading plot shows the covariance p[1] against the correlation p(cor)[1] of the variables of the discriminating component of the OPLS-DA model. Signals were assigned in each bin as follows: Bin 92, D-glucose, carnitine and betaine; bin 94 & 95, D-glucose; bin 96, choline and D-glucose; bin 97, D-glucose, glycerol and glycine; bin 103,105 & 106, D-glucose; bin 125, β-glucose; bin 135, α-glucose.

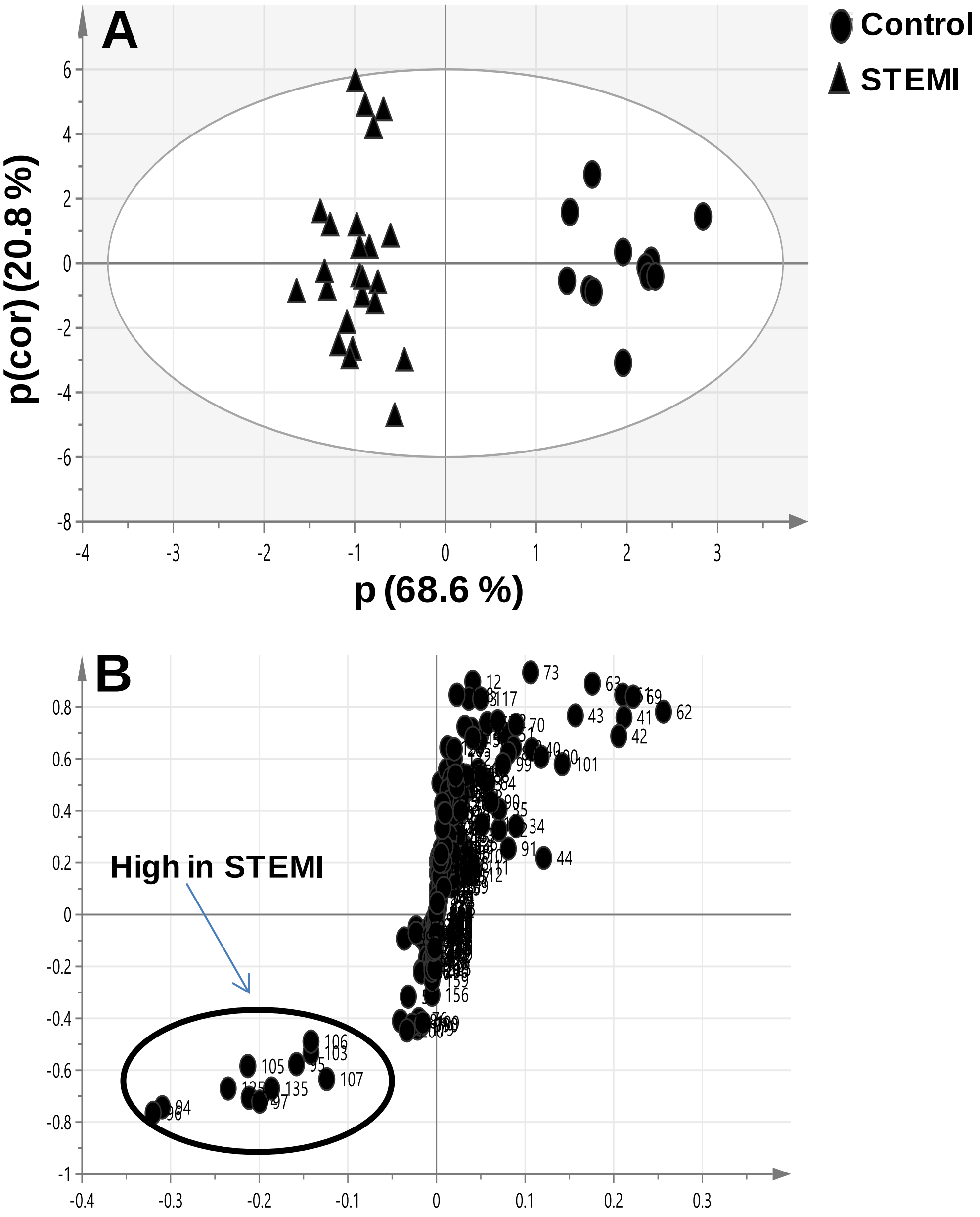

Supplement: Supplementary Information [file srep36359-s1.pdf]
